# Switching from zonisamide to perampanel improved the frequency of seizures caused by hyperthermia in Dravet syndrome: a case report

**DOI:** 10.1186/s13256-023-04307-z

**Published:** 2024-01-03

**Authors:** Kazuhiro Horiuchi, Akihiko Kudo, Shuntaro Nakamura, Kazuki Yamada, Takashi Inoue, Shintaro Fujii, Yuki Oshima

**Affiliations:** 1 Department of Neurology, Hakodate Municipal Hospital, 1-10-1, Minato-Cho, Hakodate, Japan; 2 Department of Neurology, Faculty of Medicine and Graduate School of Medicine, Hokkaido University, Kita15, Nishi7, Kita-Ku, Sapporo, Hokkaido 060-8638 Japan

**Keywords:** Dravet syndrome, Zonisamide, Status epilepticus, Perampanel, Case report

## Abstract

**Background:**

Dravet syndrome is a severe epilepsy disorder characterized by drug-resistant seizures and cognitive dysfunction, often caused by *SCN1A* gene mutations. It leads to neurodevelopmental delays and motor, behavioral, and cognitive impairments, with a high mortality rate. Treatment options include sodium valproate, clobazam, and newer agents such as cannabidiol and fenfluramine. Zonisamide, which is used in some cases, can cause hyperthermia and oligohydrosis. Herein, we present a case of a patient with Dravet syndrome whose seizures were controlled by treating infections and switching from zonisamide to perampanel.

**Case presentation:**

A 24-year-old Japanese man with Dravet syndrome presented to our department with aspiration pneumonia. The patient had been treated with valproate, sodium bromide, and zonisamide for a long time. His seizures were triggered by hyperthermia. The patient was experiencing a sustained pattern of hyperthermia caused by infection, zonisamide, and persistent convulsions, which caused a vicious cycle of further seizures. In this case, the control of infection and switching from zonisamide to perampanel improved seizure frequency.

**Conclusion:**

Dravet syndrome usually begins with generalized clonic seizures in its infancy because of fever and progresses to various seizure types, often triggered by fever or seizure-induced heat due to mutations in the *SCN1A* gene that increases neuronal excitability. Seizures usually diminish with age, but the heat sensitivity remains. In this case, seizures were increased by repeated infections, and hyperthermia was induced by zonisamide, resulting in status epilepticus. Perampanel, an aminomethylphosphonic acid receptor antagonist, decreased seizures but caused psychiatric symptoms. It was effective in suppressing seizures of Dravet syndrome in this patient.

## Background

Dravet syndrome (DS) is a severe developmental epileptic encephalopathy characterized by frequent drug-resistant seizures and cognitive dysfunction that adversely affect the quality of life and cognitive function of patients and their families (1). It is characterized by prolonged seizures, typically provoked by hyperthermia. These seizures are often caused by heterozygous loss-of-function mutations in the sodium channel protein type 1 subunit α (*SCN1A*), significantly reducing the level of the α-1 subunit of the corresponding functional protein—the neuronal voltage-gated sodium channel NaV1.1. NaV1.1 is an important central nervous system sodium channel and is highly expressed in many GABAergic inhibitory neurons [2]. When the production of this protein is inhibited, neuronal networks become hyperexcitable. Patients with DS experience neurodevelopmental delays and motor, behavioral, and cognitive impairments and have a high mortality rate primarily due to sudden epileptic death and status epilepticus (SE) [1].

Sodium valproate, clobazam, stiripentol, zonisamide, and topiramate, as well as a ketogenic diet, can be used to treat DS, and bromide is sometimes used in resistant cases. Newer agents, such as cannabidiol and fenfluramine, have been proven useful in clinical trials [3]. However, outlining prompt rescue treatment for prolonged seizures is equally important [4].

Zonisamide is a derivative of benzisoxazole with a unique chemical structure. Zonisamide acts through several complementary mechanisms, including blocking voltage-gated sodium channels (providing partial-onset seizure activity), inhibiting T-type calcium channels (providing absence seizure activity), increasing c-aminobutyric acid release, and inhibiting glutamate release [5]. Oligohydrosis has been reported in a few patients receiving zonisamide, with hyperthermia occurring in some patients [6]. Most reports of hyperthermia have been in children, typically during the summer months [7], but adult cases have also been reported [8]. However, the mechanism of zonisamide-related oligohydrosis is not fully understood. In Japan, all reported cases of zonisamide-related oligohydrosis or hyperthermia were in children, with an incidence of 1 case per 10,000 pediatric years during the first 11 years of marketing. In all cases, oligohydrosis was reversible upon zonisamide discontinuation. It has been reported that allowing children to remain cool and adequately hydrated during hot weather minimizes the possibility of hyperthermia resulting from oligohydrosis [5]. Here, we report the case of a 24-year-old patient with DS whose SE was controlled after repeated treatment for infection and initiation of perampanel instead of zonisamide.

## Case presentation

The Japanese patient first experienced myoclonus at 3 months of age generalized onset clonic seizures appeared at 6 months of age. His birth history was unremarkable, and he had no family history of neuromuscular disease, including epilepsy. Fever or hyperthermia triggered recurrent seizures, and at the age of 1 year and 5 months, the patient was diagnosed with DS at another hospital specializing in epilepsy. Valproate, sodium bromide, and zonisamide were administered. The patient’s childhood seizures were limited to generalized onset absence seizures after waking and partial seizures during sleep, and generalized onset clonic seizures were present only once a month when his body temperature was elevated, for example, during bathing. Developmental delay was observed in language, motor skills, and bulbar function. His speech was limited to single words, and the patient crawled on all four extremities and could take oral food with assistance. He had no medical history other than DS. He also had no history of drinking, smoking, or allergies and was living at home under the care of his family.

At the age of 24 years, the patient had aspiration pneumonia and fever accompanied by generalized onset clonic seizures, for which he was treated in our department for the first time. His vital signs were as follows: a heart rate of 143 beats/minute blood pressure of 123/86 mmHg, respiratory rate of 32 breaths/minute, body temperature of 39.9 °C, and SPO_2_ of 92% (room air). Jaundice in the ocular conjunctiva, swollen lymph nodes in the neck, and heart murmur were not noted. However, coarse crackles were observed in the left lung upon inspiration. Furthermore, the abdomen was flat and soft with no tenderness. He had no percussion pain in the lumbosacral region or edema in the lower extremities. No other skin rashes were observed, except for numerous acne lesions on the face. Neurological examination revealed motor aphasia, resulting in the absence of speech. His pupils were 3 mm in diameter bilaterally, his eyes were in the midline, and his light reflex was rapid. Soft palate elevation was poor and tongue movement was minimal. Muscle strength could not be measured as indicated, but the extremities could be moved by the patient voluntarily, implying no tetraplegia. Tendon reflexes were normal, and no sensory deficits or cerebellar ataxias were evident. However, he had difficulty maintaining a sitting position. Initial laboratory studies showed the following: a white blood cell count of 5200/mm^3^, hemoglobin of 15.0 g/dL, platelet count of 126 × 10^3^/ μL, C-reactive protein of 0.76 mg/dL, aspartate aminotransferase of 15 IU/L, alanine aminotransferase of 9 IU/L, blood urea nitrogen of 12.3 mg/dL, creatinine of 0.54 mg/dL, sodium of 138 mEq/L, potassium of 3.9 mEq/L, and chloride of 190 mEq/L. His blood valproic acid concentration was 80.8 μg/mL. Urinalysis revealed a urine specific gravity of 1.013 and a pH of 7.5, with no glucose, protein, fresh blood, leukocytes, or bacteria detected. Additionally, sputum culture detected *Haemophilus influenzae*. Chest X-ray showed no clear evidence of pneumonia, but chest computed tomography revealed pneumonia in the lower lobe of the left lung.

**Fig. 1:**
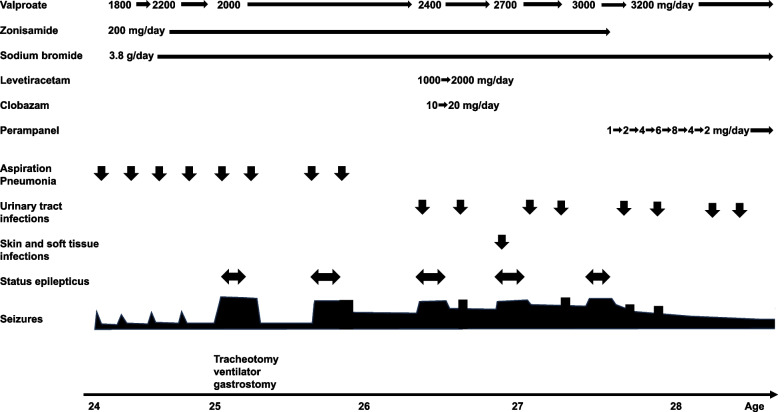
Clinical course of the patient. The patient, aged 24 years, was taking valproate, sodium bromide, and zonisamide as antiseizure medications. Seizures were triggered by aspiration pneumonia and fever. Despite treatment, dysphagia and recurrent pneumonia led to respiratory failure, necessitating intubation, tracheostomy, and gastrostomy for nutrition and medication administration. Seizures persisted, triggered by infections and elevated body temperature, resulting in status epilepticus (SE) treated with high doses of phenobarbital, midazolam, or thiopental. We suspected that the seizures and SEs may have been caused by fever due to infection and hyperthermia due to zonisamide, so we treated the infection early and discontinued zonisamide. After switching from zonisamide to perampanel, SE and seizure frequency improved

After admission, his temperature was often in the range of low-to-high 37 °C. Brain magnetic resonance imaging identified no abnormalities. The clinical course is shown in Fig. 1. His antiseizure medication (ASM) regimen consisted of 1800 mg/day of valproate, 3.8 g/day of sodium bromide, and 200 mg/day of zonisamide and remained unchanged for more than 5 years. All ASMs were orally administered. We treated the patient for aspiration pneumonia with antibiotics, although his generalized onset clonic seizures occurred during fever. Once the fever subsided, the frequency of generalized onset clonic seizures returned to that before admission. Once the fever associated with his infection resolved, the frequency of his seizures returned to his baseline level.

At the age of 25 years, the patient had pneumonia that required admission to a hospital. Pneumonia improved with antibiotics but soon worsened again, leading to respiratory failure. The patient was intubated and placed on a ventilator and underwent tracheostomy and gastrostomy. Nutrition, fluids, and medications were administered through the gastrostomy. The seizures were repeatedly triggered by a variety of factors, including fever due to urinary tract infection or skin and soft tissue infection, and high body temperature due to bathing.

Infection and seizures themselves caused a temperature elevation, sometimes to 39 °C or higher, which persisted for about 3 days. In the past, he used to have a generalized onset clonic seizure once a month, but he began to have a seizure once every 2–3 days, and sometimes every day.

The seizures caused a further prolonged temperature elevation, which led to the appearance of SE. Gradually, the patient’s SE became more prolonged, and the hospitalization period became longer. In SE, diazepam alone was insufficient to control persistent seizures, and a single dose of high-dose phenobarbital or continuous administration of midazolam or thiopental was often required. The cause remains unclear; perhaps, it could be the frequent administration of antimicrobials, the change from oral to tube feeding, or the resulting reduced absorption of valproic acid from the gut. Notably, blood valproic acid levels continued to decrease after antimicrobial therapy was discontinued. Therefore, the dose of valproate had to be gradually increased with the measurement of the blood levels.

**Fig. 2:**
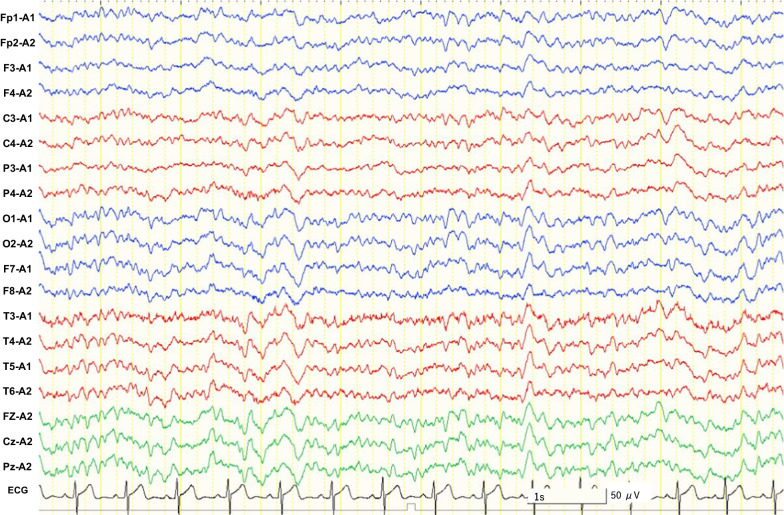
Interictal electroencephalogram (EEG). Interictal EEG showed a mixture of sharp slow-wave complexes and sharp waves. EEG displayed at 7 µV/mm, HFF 70 Hz, and LFF 1 Hz. Calibration bars equal 50 μV vertical and 1 s horizontal

An electroencephalogram (interictal phase) is shown in Fig. 2. We considered the possibility that fever due to repeated infections and repeated seizures, as well as the side effects of zonisamide, may have contributed to the high body temperature. We explained to the family that we were discontinuing zonisamide because it might be contributing to the high body temperature. However, the family did not agree to discontinue zonisamide, which had been used for a long time for the patient.

Clobazam and levetiracetam were added but were discontinued without the effect on seizure control. The patient continued to have SE each time he had hyperthermia. The symptoms tended to worsen, and given that symptoms have been reported to worsen with hyperthermia in DS associated with an *SCN1A* gene mutation, the patient underwent genetic testing (with the family’s consent) to confirm that the symptoms were caused by DS and not other diseases. Genetic testing revealed a frameshift mutation (c.2176 + 1G>A) in the *SCN1A* gene. Based on the course and findings, the diagnosis was consistent with DS.

During aspiration pneumonia, the patient experienced poor oxygenation with fever, coarse crackles in the lungs on auscultation, and an increased sputum volume. After respirator attachment, the airway pressure sometimes increased because of sputum accumulation. Sputum culture often detected *Pseudomonas aeruginosa* and *Corynebacterium striatum*. For antimicrobial therapy, the patient received 4.5 g of intravenous tazobactam/piperacillin four times a day or 500 mg to 1 g of intravenous vancomycin twice a day (changed according to blood concentration) for 14 days. If no increase in sputum production or poor oxygenation occurred, a urinary tract infection was considered. Urinalysis revealed urinary bacteria, and no other infections were considered. In urine cultures, *Klebsiella oxytoca*, *Escherichia coli* (sometimes extended-spectrum β-lactamase-producing bacteria), and *P. aeruginosa* were found. The above-mentioned dosage of intravenous tazobactam/piperacillin also addressed urinary tract infections. When pneumonia and urinary tract infections could be treated in the outpatient setting, 500 mg of oral levofloxacin was administered once a day for 14 days. Urinary tract infections, pneumonia, and skin infections were treated with antibiotics as early as possible to prevent the occurrence of fever. At the age of 27 years, SE due to hyperthermia continued without infection, and administration of thiopental was continued for 2 weeks in the hospital. With the family’s consent, zonisamide was tapered off, and perampanel was titrated up from 1 mg/day. Repeated episodes of impetigo all over his body were treated with topical antimicrobials each time. Oral minocycline was administered for severe acne. After discontinuing zonisamide in the outpatient setting and switching to perampanel, the patient’s hyperthermia and associated seizures resolved in a short period, occurring only once a week, due in part to the effect of less frequent fever caused by infection and perampanel. His temperature was also in the 36 °C range (previously, his body temperature was often in the 37 °C range), possibly due to the discontinuation of zonisamide.

One and a half years after the discontinuation of zonisamide and switching to perampanel, SE no longer occurred; however, mild seizures continued at least once a week. Perampanel was titrated up to 8 mg/day; however, the patient became mentally restless and attempted to remove the ventilator by hand because of spontaneous movements. We reduced the dose of perampanel to 2 mg/day. No worsening of seizure frequency (once a week) was observed after reducing the dose of perampanel. Furthermore, 3200 mg/day of valproate and 3.8 g/day of sodium bromide were continued. The patient was in a state of total bed rest with no speech, and although he could raise his upper extremities, the lower extremities were almost completely motionless because of disuse. He could no longer swallow.

## Discussion and conclusion

The patient had DS, a condition in which high fever results in seizures. He also had recurrent SEs resulting from fever caused by aspiration pneumonia and a urinary tract infection. Zonisamide may have contributed to the hyperthermia, and a change in the antiepileptic drug from zonisamide to perampanel prevented SEs and reduced seizures. Although zonisamide may cause hyperthermia, no reports have shown that zonisamide-induced hyperthermia can cause seizures in patients with DS.

The typical presentation of DS is characterized by fever-induced generalized clonic seizures in the first year of life, followed by myoclonic, absence, focal, and generalized tonic–clonic seizures. Mutations in the *SCN1A* gene inhibit GABAergic inhibitory interneurons, causing neuronal hyperexcitability. Seizures in patients with DS are usually initially triggered by fever or other causes of body temperature elevations. Furthermore, it has been suggested that heat production from the seizures themselves induces additional seizures. Regarding the temporal evolution of epilepsy, the frequency and severity of seizures tend to decrease from adolescence to adulthood. Thermal sensitivity persists; however, its effects have been reported to decrease. The most common seizure type in adulthood is generalized tonic–clonic seizures, which occur primarily during sleep [2].

In this case report, a frameshift mutation in the *SCN1A* gene was found, and the patient had recurrent seizures with fever and hyperthermia. He had a progressive deterioration of physical functions with dysphagia and repeated aspiration pneumonia. The number of seizures gradually increased each time a fever occurred due to pneumonia or other infectious diseases. It is possible that the fever triggered by the infection was concomitant with the continued hyperthermia induced by zonisamide, resulting in persistent thermosensitive effects and induction of SE.

Perampanel is a selective, noncompetitive antagonist of the aminomethylphosphonic acid (AMPA) glutamate receptor. Numerous studies have demonstrated its efficacy and tolerability in patients over 12 years of age with focal-onset seizures, focal–to–bilateral tonic–clonic seizures, and primary generalized tonic–clonic seizures. Moreover, recent studies have reported that perampanel is effective for treating myoclonic and absence seizures in patients with genetic generalized epilepsy, intractable myoclonic epilepsy in patients without genetic generalized epilepsy, and those with specific epilepsy syndromes, including Lennox–Gastaut syndrome and some kinds of progressive myoclonus epilepsy. A few recent studies have investigated its use in patients with DS, yielding promising results; however, the number of reported cases is still limited [9].

The main reason for the reduction of SE and seizures in this case was thought to be the early treatment of the infection, which reduced the incidence of hyperthermia, as well as the fact that zonisamide had increased body temperature, a situation that could have easily induced SE and seizures in this patient. The persistence of hyperthermia decreased after discontinuation of zonisamide. However, we believe that perampanel also had an effect, because the patient, who had been suffering from recurrent SE until then, no longer had SE after starting perampanel. The incidence of psychiatric side effects of perampanel was similar between perampanel monotherapy/first adjunctive therapy (7.1–37.0%) and perampanel adjunctive therapy (21.0–24.4%) [10]. Our patient also became restless and started attempting to get off the ventilator; therefore, we reduced the dose of perampanel from 8 mg/day to 2 mg/day.

Patients and their families were able to receive home care with more feelings of relief after switching from zonisamide to perampanel because they no longer had SEs.

We will consider ASMs that are known to be effective in DS, such as stiripentol or fenfluramine [11], if the frequency of seizures or the occurrence of SE increases.

DS is characterized by seizures triggered by fever due to infection or hyperthermia due to various other causes. In case of repeated seizures with DS, clinicians should consider treating infections early and discontinuing zonisamide if a high body temperature results from zonisamide. In our case presented here, perampanel was useful in controlling seizures in DS.

## Data Availability

The datasets used and analyzed during the current study are available from the corresponding author on reasonable request.

## Abbreviations

DS: Dravet syndrome
SE: Status epilepticus
ASM: Antiseizure medication
SCN1A: Sodium channel protein type 1 subunit α
AMPA: Aminomethylphosphonic acid
